# Diseases of Infants

**Published:** 1881-07

**Authors:** C. Lippe

**Affiliations:** New York


### DISEASES OF INFANTS.

C. Lippe, M. D., New York.

For the selection of the proper homœopathic remedy in diseases of infants, we have only the symptoms observed in the patient for our guide; and at times, by those symptoms we can discover the kind of pain and the locality.

First is to be considered the countenance, for by the wrinkles, lines, color and expression the attentive observer ascertains often the anatomical seat of the trouble. Wrinkles and lines on the forehead lead us to suspect brain difficulties, between the forehead and upper lip, chest troubles, and about the chin, abdominal complaints.

Wrinkles on the forehead call our attention to *Cham.*, *Hell.*, *Lyc. Rheum.*, *Rhus.*, *Sepia.*, *Stram.:* Knitting the brows, to *Rheum.*, *Viol-od.* Wrinkles on the face, to *Calc.*, *Hell.*, *Lyc.*, *Stram.;* on the lips, to *Amm-c.* Blueness of the nose *Hydroc-acid;* paleness around the nose, *Cina.* Fan-like motion of alæ nasi, *Lyc.*

Aged expression *Hydroc-ac.*

Anxious expression *Aeth.*, *Bell.*, *Cupr.*

Bewildered expression *Plb.*, *Stram.*, *Zc.*, etc.

The sudden contraction of the muscles of the face and the quick passing expression of pain will give us an indication of a short, sharp, shooting or stitching pain; if the trouble is related to the chest, the pain will be of the stitching variety; if to the abdomen, shooting; this kind of pain is accompanied by a quick, loud cry, if the infant has not been much weakened by the disease; the weaker the patient becomes, the cry is at first hoarse, then almost extinct. If the child moans constantly, the pain is more of an aching or of a dull character; if the child only manifests pain on being moved or touched, then we may safely say that the pain is of the bruised variety; *Arnica*, etc. Pain only on voluntary motion will lead us to suspect rheumatic pains, and call our attention to *Bry.*, etc.

The position the child assumes both when asleep and awake, and the gestures often also give us a clew to the kind of pain and the locality.

The upright position of dyspnœa, calling attention to the peculiar conditions requiring such a position, and the position on the side with legs flexed and arms drawn close to or over the chest, which is observed in the last stages of brain affections; one leg stretched out, the other bent; hands above the head, or crossed on the abdomen, all calling attention to the peculiar symptoms belonging to certain remedies which often are of importance in many cases.

The desire to be carried and amelioration from being so carried has so often been observed to be met by *Cham.*, that this symptom has been marked as characteristic under that drug, and in croup the necessity of being carried with a rapid motion has been observed under *Brom.*

The continuous crying of infants may be referred to two reasons, earache or hunger, when not due to mechanical causes.

The peculiar cries are of some importance. The sharp, shrill, single cry of *Apis;* the moaning of *Acon.*, *Bell.*, *Hell.*, etc.; the groaning of *Millef;* whining, *Apis;* the hoarse metallic sound of croup, *Bell.*, *Brom.*, *Hepar*, *Spongia.*

The tongue gives some peculiar diagnostic signs of the remedy to be applied; black, *Ars.*, *China*, *Merc.*, *Phosp.*, etc.

Bluish, *Dig.*, *Mur-ac.*, *Spig.;* Grayish, *Ambra*, *Tart.*

Red all over, *Bell.*, *Tart.*, *Ars.*, *Lach.*, *Rhus.*, *Sulph.*

Red glistening, *Glon.*, *Kali-b.*, *Lach.*

Red, trangular tip, *Phyt.*, *Rhus.*

Red tip, undefined borders, *Rhus.*

Lead colored, *Ars.*

White coat on one side, *Rhus;* on both sides, *Caust.*

White coat, middle only, *Bry.*, *Phosp.*

White coat on the root strongly marked, *Sepia.*

Dry red cracked at tip, *Kali-b.*, *Lach.*, *Rhus.*, *Sulph.*

Dry tongue without thirst, *Bry.*, *Puls.*

Soft, with prints of teeth, *Iod.*, *Merc.*, *Rhus.*, *Stram.*

We are always to bear in mind that the Law of cure, as developed and expressed by Hahnemann, must hold good with infants as with adults, and although the selection of the proper remedy is more difficult with infants, yet from their quick rallying powers, we have made our most brilliant and rapid cures. We all know the almost instantaneous effect of a single dose of the proper remedy in croup. Our only success can be through the strict adherance to that single law, which unerringly guide every one who rigidly adheres to it.

